# Integration of a rapid scanning technique into THz time-domain spectrometers for nonlinear THz spectroscopy measurements

**DOI:** 10.1063/1.5080653

**Published:** 2019-03-25

**Authors:** C. Hoberg, P. Balzerowski, M. Havenith

**Affiliations:** Lehrstuhl für Physikalische Chemie II, Ruhr-Universität Bochum, 44780 Bochum, Germany

## Abstract

We have implemented a rapid scanning technique into THz time-domain spectrometers using an oscillating frictionless delay line, especially adapted for nonlinear THz experiments. Thereby we were able to increase the dynamic range of THz measurements in the frequency range from 40 to 200 cm^-1^ by up to 24 dB and reduce the scanning time by up to a factor of 200. We report here test measurements on TDS-setups at repetition rates of 80 MHz and 5 kHz. The dynamic range exceeds 64 dB, which allows to record even small changes in the THz absorption upon optical excitation by a THz probe, covering the frequency range of the intermolecular modes and the phonon bands. We demonstrate the potential of this technique for optical-pump THz-probe experiments using a 70 μm thick high-resistivity silicon, excited by 400 nm, ∼50 fs pulses as a sample.

## I. INTRODUCTION

THz time-domain spectroscopy (TDS) has developed into a powerful spectroscopic method for linear and nonlinear THz spectroscopy in the frequency range from 0.3 to 10 THz.^1,2^ Conventional TDS setups are based on ultrafast laser sources. A THz emitter generates few- to half-cycle ultrashort THz-pulses which are guided through the sample and detected coherently and in the time-domain.^1,2^ Typical emitters include biased photoconductive antennas and nonlinear crystals. In biased photoconductive antennas, the ultrashort laser pulse generates carriers in a semiconductor material (typically LT-GaAs), which are subsequently accelerated by an applied bias field. Alternatively, nonlinear crystals (e.g. ZnTe, GaP, LiNbO_3_) are used to generate THz pulses by optical rectification. For detection, the THz pulse is overlapped spatially and temporally with parts of the optical laser pulse either in an ultrafast optical switch or a nonlinear crystal. In the latter, the THz field induces a birefringence which is directly proportional to the amplitude of the THz field which is sampled by an ultrashort optical probe pulse. By varying the delay between the THz and the detection pulse, the amplitude of the electromagnetic field is recorded as a function of delay time. The delay time between the THz pulse and the probe pulse is controlled by varying the path length between the THz and detection pulse, conventionally using mechanical delay lines. While mechanical delay lines can achieve high precision movements, the disadvantage is its inherent slow operation. Scanning the THz pulse waveform with satisfactory resolution, time-window and integration time, typically takes up to several seconds or even minutes. Recent advancements in the field of nonlinear THz spectroscopy urge for alternatives in scanning procedures.^3,4^ More recently, so-called “single-shot” detection techniques have been developed, where the complete THz waveform is recorded for each laser shot.^4–6^ While these techniques are promising, they require a) a multitude of components and large modification of existing setups and b) typically suffer from distortions in the acquired waveform compared to classical step-scanning.

Here we report a novel rapid scanning technique based on a rapidly oscillating frictionless delay line by which scanning speeds between 0.5 Hz - 20 Hz are routinely achievable. This results in an increase in the dynamic range by up to 24 dB compared to step scanning (integration time was set to 105 s). The dynamic range exceeding 64 dB opens the way to highly sensitive frequency-resolved optical-pump THz-probe measurements.

So far, rapid scanning has been utilized with different approaches. In the first type of approach techniques avoid the use of slow mechanically moving parts, but instead rely on synchronization of two laser cavities or on the synchronization of a laser cavity with an acousto-optic delay. Examples are ASOPS (asynchronous optical sampling),^7^ OSCAT (optical sampling by cavity tuning),^8^ and AOPDF (acousto-optic programmable dispersive filter).^9^ In the ASOPS technique two femtosecond lasers operating with slightly detuned, yet locked repetition rates are used to do fast time domain scanning rate with a tunable difference.^7^ In the OSCAT technique a single laser cavity is used. Two subsequent pulses from the same cavity are used for generation and detection. To compensate the timing offset between two succeeding pulses, one pulse is delayed by a fiber.^8^ To keep the fiber length small enough a high repetition rate is required. Both techniques require specially designed laser cavities and operate at high repetition rates (80-1000 MHz) only. The AOPDF technique utilizes an acousto-optic programmable dispersive filter. Inside the filter the optical pulse train co-propagates with an acoustic wave in a birefringent crystal. The group velocity of the optical pulse exceeds the acoustic wave and therefore successive pulses will reach the acoustic wave at a slightly different positions. At each of these positions the pulse will switch its polarization to the extraordinary axis. Thus, every pulse will acquire a different time delay corresponding to the interaction point with the acoustic wave.^9^

Alternatively, mechanical devices are used to vary and control the time delay between the two pulses: Previously, techniques involving rotational delay lines (RODL) were reported utilizing a helicoid surface or a convex reflective surface.^10–12^ Alternatively, oscillating optical delay lines (OODL) were used.^13,14^

All aforementioned techniques were demonstrated for high repetition rate laser systems with low pulse energies only (ranging from 11 MHz up to 1 GHz with pulse energies less than 9 nJ). All techniques were designed with the intention of reaching very high scan rates and/or high frequency resolution.

However, for non-linear experiments high E-fields are mandatory. This requires the use of amplified laser systems which generate ultrashort, high-energy pulses (1-10 mJ and more). Typically, these laser systems are operated at low repetition rates of 1-10 kHz. Whereas previously reported rapid scanning techniques focused on high repetition laser systems we focus here on an alternative technique which is compatible with both, high and low repetition rate laser systems.

Our concept is based on a frictionless linear delay line that can be operated with triangular instead of sinusoidal movement, thus the spacing between successive laser pulses is kept constant. Furthermore, the precise position of the mirror is synchronously recorded with a photo-electric sensor. In this way, no calibration or interpolation of the delay line movement (as used in previous techniques) is needed thus reducing the positioning uncertainty.^15^ In addition, a high frequency digital lock-in amplifier or boxcar integrator is used for low-frequency noise reduction.

Here, we demonstrate that this rapid scanning technique can be applied in THz time-domain systems with low as well as high laser repetition rates while improving the dynamic range by up to 24 dB. Finally, we demonstrate that this rapid scanning technique can be applied to nonlinear THz techniques that utilize more than one delay line, such as optical-pump THz-probe experiments (OPTP) to reduce scanning times by a factor of 20 or more.

## II. EXPERIMENTAL RESULTS

### A. Integration of rapid scanning techniques in MHz repetition rate systems

**FIG. 1.**
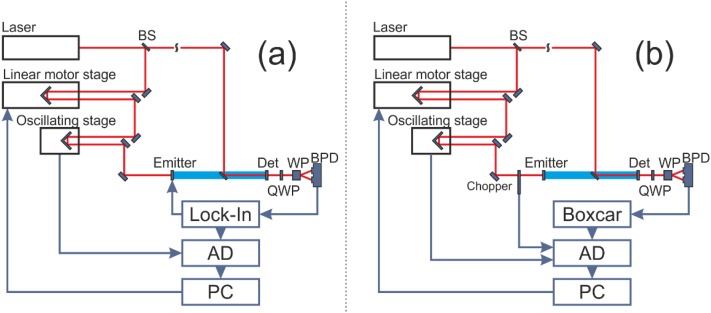
Setup of the rapid scanning THz time domain spectrometers using (a) a Ti:Sa-oscillator (80 MHz repetition rate), (b) an amplified Ti:Sa system (5 kHz repetition rate) as radiation sources. BS: beam splitter, QWP: quarter wave plate, WP: Wollaston prism, BPD: balanced photodiodes.

An overview of the TDS setup including a high repetition rate laser (such as standard, non-amplified Ti:Sa oscillators with repetition rates in the MHz range, here: MaiTai HP, SpectraPhysics) is given in Fig. 1. In short, each ultrashort pulse is split into a pump and a probe pulse. The pump pulse is used to generate a THz pulse in an antenna. The transmitted THz pulse induces birefringence in the detector crystal (ZnTe<110> crystal) which is probed by measuring the induced polarization change of the probe pulse.

The probe pulse is delayed with respect to the pump pulse by motorized linear stages. To scan the THz pulse a step scan is performed: the linear translation stage is moved by a step size distance Δx, yielding a delay of Δt in the optical pathway. The signal of the balanced diodes is recorded with an integration time t_int_. For the rapid scanning approach, we included an oscillating frictionless delay line (scanDelay 50, APE GmbH). The accessible scan range is 50 ps (7.5 mm) with scan frequencies between 0.1 Hz and 20 Hz.

To record the THz pulse, the oscillating delay line is set to a fixed speed and scan range. In contrast to previously reported studies, we do not synchronize the oscillating stage to an external trigger. Instead, the position of the stage is continuously recorded by means of an internal analog photo-electric system.

The balanced photodiode signal is amplified by means of lock-in amplification (HF2LI, Zurich Instruments). For amplitude modulation we directly modulate the bias of the THz antenna.

Due to the rapid-scanning, the lock-in must be set to a small time-constant, so that it reacts fast enough to the signal changes. If the time-constant is too large (and therefore its bandwidth too small), the bandwidth of the recorded waveform would be reduced. We use high modulation frequencies (>100 kHz) and small time constants (<100 μs) for optimal SNR. The position signal of the oscillating delay and the demodulated diode signal are synchronously digitized and evaluated in real-time.

**FIG. 2.**
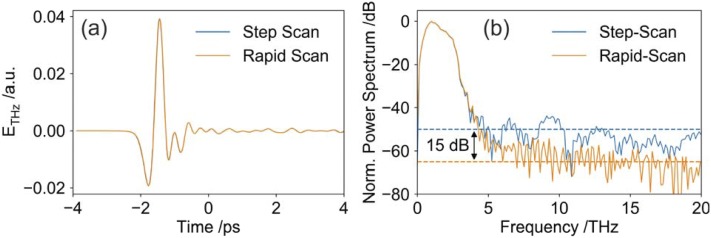
(a) Comparison of THz Time-Domain traces recorded with rapid scanning (orange, 5 s integration time) and step-scanning (blue, 990 s recording time) demonstrating that both waveforms match. (b) Corresponding normalized power spectra. For the rapid scan the dynamic range is improved by 15 dB.

In the following, we compare the normal step-scan mode with our rapid scanning technique. For comparison, we recorded a THz pulse with each method and compare the respective Fourier transforms (Fig. 2).

First, the step-scan was recorded (10 fs resolution, 8 ps window, 500 ms integration per point). The integration time was 400 s, however, the mechanical delay line required additional time to reach the set position. Therefore, the total recording time was 990 s.

Next, we recorded the same time-domain trace (identical resolution and time window) using rapid scanning technique. We chose a scanning rate of 4 Hz and integrated over 20 individual scans, which required a total recording time of 5 s. Despite of the decreased recording time (rapid scanning was nearly 200x faster), we were able to improve the dynamic range by 15 dB.

### B. Integration of rapid scanning in KHz repetition rate systems

**FIG. 3.**
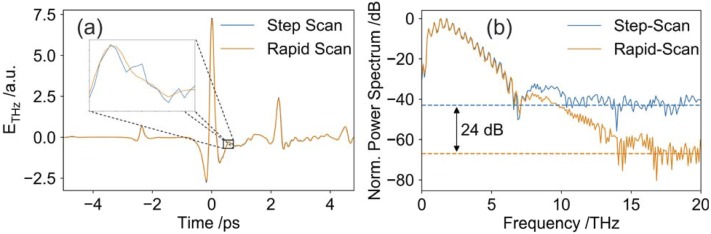
(a) THz Time-Domain traces recorded with rapid scanning (orange) and step-scanning (blue). Both waveforms match perfectly, however, the inset shows superior SNR for rapid scanning. (b) Corresponding normalized power spectra. The dynamic range for the rapid scan is increased by 24 dB compared to the step-scan.

In Fig. 1(b) we show a rapid scan setup for an amplified Ti:Sa source with repetition rates in the kHz range (1-10 kHz). In this case, we did not use a lock-in detection scheme. Instead, the signal of the balanced diodes is recorded individually for each laser pulse by means of boxcar integration (UHFLI, Zurich Instruments) which allows for a much faster sampling time. We use an oscillating delay line with an accessible scan range of 15 ps (scanDelay 15, APE). By optically chopping the pump pulse, THz and background signals are recorded, and the difference is deduced. In our case, the resulting data acquisition rate is 2.5 kHz (equivalent to 400 μs). A 10 ps scan with a temporal resolution of 20 fs consists of 500 data points. We want to point out, that a complete time-domain scan can be recorded in 200 ms (5 Hz). As can be seen in Fig. 3, for the rapid scan, the dynamic range is increased by 24 dB for the same integration time (105 s).

The reason for the improvement in performance can be explained in the following way: For a typical THz time-domain system, there are several noise contributions to consider. The relative significance of these noise sources may vary for different systems. Often, the main source of noise in the THz time-domain setup is directly related to the laser stability such as energy-, pointing- and polarization stability. For amplified laser systems, especially the (pulse-to-pulse) energy stability and power drifts are often critical. Other sources of noise include

• noise related to the sample (e. g. thickness variations over time, changes in humidity, temperature variations, …)
• noise related to the setup (e. g. vibrations, temperature of nonlinear crystals, jitter of optical choppers, positioning errors of delay stages etc.)
• noise related to detection (e. g. shot noise)
• noise related to electronics (however typically to a much lesser extent)

Typically, these (often systematic) noise contributions are dominant in the low frequency spectrum, because they correspond to slowly varying changes or drifts (e. g. over the course of seconds or longer). The standard step-scanning procedure is inherently slow. Hence the low-frequency noise contributions cannot be sufficiently suppressed. In our presented approach, scanning the THz pulse quickly (and more often), will shift the signal of interest (the detected THz signal) to higher frequencies, thus reducing the noise level.

Another reason for better performance: With the step-scanning procedure, the mechanical delay line needs additional time to reach the next position. This means there is always some “dead time” between two measurement points. Within this “dead time” no data points are acquired. Effectively these data points are lost and do not contribute to the scan. The duration of the “dead time” depends on the specific type and model of delay line, but typically lies in the range of 100-500 ms per point and can easily make up 50% of the total measurement time. On the other hand, in the rapid-scanning approach each laser shot is used, since the position of the stage is always known. Therefore, significantly more data points are recorded and thus a better SNR ensues.

## III. INTEGRATION OF RAPID SCANNING TECHNIQUES FOR NONLINEAR THz SPECTROSCOPY

In general, nonlinear THz spectroscopies, such as optical-pump THz-probe spectroscopy (OPTP) or two dimensional THz spectroscopy, use more than one optical delay line.^16^ In OPTP experiments, the difference of the transmitted THz field with and without optical excitation is recorded as a function of both delay lines (THz-probe delay time and pump delay time). As a first application, we integrated the rapid scanning technique into the THz-probe delay line (while keeping a motorized linear delay line for the pump). For a proof of principle experiment we used a 70 μm thick high-resistivity silicon wafer (HR Si) as a sample, which was excited by an optical pump pulse at 400 nm. Silicon is an indirect semiconductor with a bandgap of 1.12 eV at room temperature.^17^ In the spectral range below 10 THz, high-resistivity (<10 kΩ cm) silicon has an absorption coefficient of less than 10 cm^−1^.^18^

**FIG. 4.**
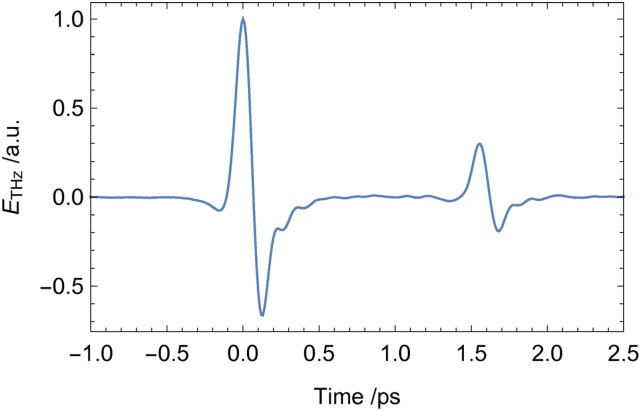
Time-domain waveform of the THz pulse transmitted through a 70 μm thin high-resistivity silicon wafer. The second pulse corresponds to the first internal reflection within the silicon wafer.

Fig. 4 depicts the THz waveform transmitted through a 70 μm thin sample of high-resistivity silicon. Upon photo-excitation above the band gap of 1.12 eV (λ = 1.107μm), free carriers are generated, which increase the conductivity of the semiconductor.^19^ This increased conductivity causes a Drude type absorption, which results in a nearly complete opacity of the photoexcited semiconductor in the THz spectral range, which can be directly observed in OPTP.

**FIG. 5.**
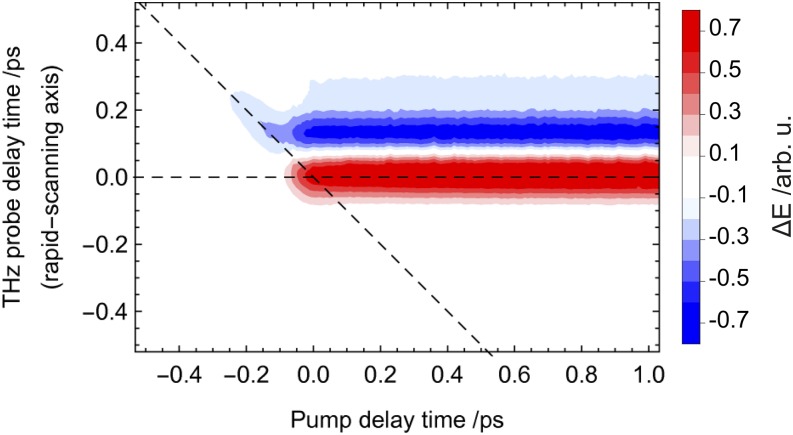
Optical-Pump THz-Probe scan of 70 μm thick HR-Si, excited by 400 nm, ∼50 fs pulses. The time resolution along both axes is 10 fs. The recording time was 15 min for the complete map. The color bar is normalized to the difference in E-field w.r.t. the (unpumped) THz peak E-field.

For the OPTP scan, we chose a time step of 10 fs on both axes and a scan-window of 1.5 ps (pump) by 2 ps (THz probe). The complete map was recorded in 15 min, more than 20 times faster compared to a scan using two motorized delay lines. The result is displayed in Fig. 5.

The photo-excitation response can be modeled as a convolution of a Heaviside step function H(t), corresponding to the instantaneous electronic response of silicon, and the temporal width of the pump pulse I_pump_ and the EOS sampling pulse I_detection_. Using Gaussian temporal profiles, the pump and probe pulse functions is combined to yield an effective response. The full response R(t) is proportional to an error function defined as:

$$\begin{array}{ccc} \begin{array}{c} R\left(t\right)=H\left(t\right)I_{pump}I_{detection}\\ R\left(t\right)=H\left(t\right)I_{eff}\\ \Rightarrow R\left(t\right)\propto \frac{1}{2}\left(1+erf\left(\frac{t-t_{0}}{\sqrt{2}\sigma_{eff}}\right)\right) \end{array} & & \end{array}$$ (1)

**FIG. 6.**
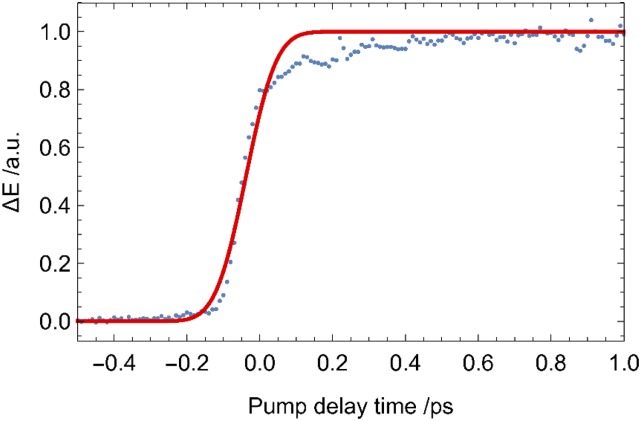
One dimensional cut along the dashed line parallel to the pump-delay axis of the OPTP data set depicted in Fig. 5. The solid line corresponds to the fit according to Eq. (1).

Fig. 5 shows a contour plot of the OPTP data set obtained for a 70 μm thin silicon wafer using 400 nm pump pulses with a pulse energy of 16 μJ. For the measurement, a small piece of the wafer is placed at the sample position. The data set consists of the difference between the THz waveforms, which are recorded with (E_on_(t_THz_)) and without (E_off_(t_THz_)) optical pumping at each pump-probe delay position. This reflects the difference of the detected THz waveform as a function of the pump pulse arrival (∆E(t_pump_, t_THz_)). The signal amplitude is calculated as the relative change with respect to the absolute maximum of the detected THz waveform with the pump pulse off (E_off_(t_THz_)). Positive values of t_pump_ correspond to a pulse order in which the 400 nm pump pulse arrives first at the sample position. Fig. 6 depicts the one dimensional cut along the horizontal dashed line of Fig. 5 parallel to the t_pump_ -axis. This cut corresponds to the change of the peak of the transmitted waveform upon photo-excitation. The fast dynamic change directly reflects the increased absorption due to the generated free carriers.

Fitting the observed one dimensional photoexcitation response in Fig. 6 to Eq. (1) results in an effective time resolution of σ_eff_ = (65.6 ± 0.3) fs. The slight deviation from the fit, which assumes a perfect collinear alignment of optical pump an THz probe, indicates a small deviation from collinearity that could also be attributed to the response function of the GaP detection crystal itself.

## IV. SUMMARY

We present a novel, rapid scanning technique especially designed for non-linear THz experiments using amplified laser systems with low repetition rates. This approach utilizes a frictionless continuously oscillating delay line, thereby reducing the scanning time considerably while increasing signal to noise. We report here an increase in the dynamic range by 24 dB for a TDS System with a 5 kHz repetition rate and an increase of 15 dB in a laser system with MHz repetition rate. While this technique is also widely applicable in linear THz spectroscopy, recent advancements in the field of nonlinear-THz spectroscopy show the need for alternative scanning procedures.^3,4^ The potential of optical-pump THz-probe spectroscopic measurements for studying hydration dynamics of solvated chromophores, seen from the perspective of the solvent, have been demonstrated recently by the Hamm group.^20^ The reported decrease in scan times going along with an increase in dynamic range will allow to further push the development of sensitive multidimensional THz techniques.
